# Cryo-EM structures of apo and atorvastatin-bound human 3-hydroxy-3-methylglutaryl-coenzyme A reductase

**DOI:** 10.1107/S2053230X25001098

**Published:** 2025-02-20

**Authors:** Manikandan Karuppasamy, Jason van Rooyen

**Affiliations:** a eBIC-for-Industry Diamond Light Source Harwell Science and Innovation Campus DidcotOX11 0DE United Kingdom; University of Leeds, United Kingdom

**Keywords:** SPA cryo-EM, HMGR, atorvastatin, graphene oxide

## Abstract

The enzyme 3-hydroxy-3-methylglutaryl-coenzyme A reductase (HMGR) regulates the level of cholesterol by catalysing the formation/production of mevalonate and has therefore become an important pharmaceutical target for coronary heart disease. Here, we report the cryo-EM structure of the catalytic part of the enzyme in the apo form and bound with its inhibitor atorvastatin, a commonly used drug in cardiovascular disease, at resolutions of 2.1 and 2.3 Å, respectively. In the cryo-EM maps, part of the N-domain corresponding to amino acids 439–487 is well ordered and could be modelled completely. Atorvastatin molecules were found to occupy all four active sites of the tetrameric complex, and the binding does not alter the conformation of the protein or the active site. The method described here exploits graphene oxide as an additional support and could be used as an alternative to elucidate the structures of pharmaceutical target compounds that are difficult to co-crystallize with human HMGR and for sparsely available samples in drug discovery.

## Abstract 2

Single-particle cryo-EM structures of apo and atorvastation-bound human 3-hydroxy-3-methylglutaryl-coenzyme A reductase were determined using graphene oxide-coated grids.

## 1. Introduction

A high level of low-density lipoprotein in plasma cholesterol was found to be responsible for coronary heart disease in the 1950s (Tobert, 2003 ▸). The rate-limiting enzyme 3-hydroxy-3-methylglutaryl-coenzyme A (HMG-CoA) reductase (HMGR) catalyses the reduction of HMG-CoA to mevalonate (Istvan *et al.*, 2000 ▸) and tightly regulates the level of cholesterol in humans. Therefore, the human enzyme (hHMGR) has been identified as a potent natural target for hypercholesterolemia diseases resulting from higher levels of cholesterol (Gesto *et al.*, 2020 ▸). A family of inhibitors commonly known as statins have been found and used as drugs to reduce cholesterol levels (Endo & Hasumi, 1993 ▸; Tobert, 2003 ▸; Endo, 2010 ▸). The crystal structures of tertiary complexes of the enzyme, substrate and different statin inhibitors have been solved to understand the detail of the molecular mechanism of the mode of inhibition (Istvan *et al.*, 2000 ▸; Istvan & Deisenhofer, 2001 ▸; Bose *et al.*, 2023 ▸; Vögeli *et al.*, 2019 ▸; Haywood *et al.*, 2022 ▸). The statin molecule partly occupies the binding site for the substrate HMG-CoA and thereby blocks its access to the active site (Istvan *et al.*, 2000 ▸; Istvan & Deisenhofer, 2001 ▸).

Electron cryogenic transmission microscopy with single-particle analysis (SPA cryo-EM) has become an inevitable alternative method for deciphering the three-dimensional structures of biological molecules in their near-native state (Henderson, 2015 ▸; Russo *et al.*, 2022 ▸; Chari & Stark, 2023 ▸). The technique has helped to elucidate several distinct structures of protein complexes, in particular membrane proteins that are difficult to crystallize (Harrison *et al.*, 2023 ▸). The number of structures deposited in the Protein Data Bank (PDB; Berman *et al.*, 2000 ▸) using SPA has been increasing exponentially (EMDB; wwPDB Consortium, 2024 ▸; Russo *et al.*, 2022 ▸).

In the X-ray diffraction technique, soaking and co-crystallization are the two common approaches for studying protein–ligand, enzyme–inhibitor and protein target–drug molecule interactions (Hassell *et al.*, 2007 ▸; Wienen-Schmidt *et al.*, 2021 ▸). However, most pharmaceutical compounds require organic solvents such as DMSO to dissolve them. This process generally inhibits co-crystallization, and protein crystals often crack when soaked due to the diffusion of such molecules through the crystal solvent channels. In addition, for soaking to be successful the binding site needs to be accessible and not blocked by the protein crystal contact interactions. These approaches reduce the chances of obtaining the 3D structure of macromolecules with small molecules by X-ray diffraction analysis. Therefore, single-particle cryo-EM can be a complementary technique as high-resolution structures can be obtained without growing crystals as well as in the presence of a relatively low percentage (≤10%) of organic solvents that are compatible with target protein and vitrification process.

Here, we used SPA cryo-EM to solve the structures of the apo and atorvastatin-bound forms of the catalytic domain of the important pharmaceutical target hHMGR at atomic resolution by exploiting graphene oxide as an additional support on the holey carbon grids. To our knowledge, the apo structure of the human enzyme has previously not been reported; we describe the structure at 2.1 Å resolution and compare it with the inhibitor atorvastatin-bound form.

## 2. Methods

### 2.1. Sample

The recombinantly prepared catalytic domain of human HMGCoA reductase, Ser426–Ala888 (molecular weight 51 kDa), was purchased from Bio-Techne, USA (product code 9264-MH-020). The enzyme was at a concentration of 0.214 mg ml^−1^ in 25 m*M* Tris pH 7.5, 150 m*M* NaCl, 5 m*M* DTT, 50%(*v*/*v*) glycerol and included an N-terminal methionine residue and a His_6_-tag. The inhibitor atorvastatin was purchased from Cambridge BioScience (product code 2278-10) and a stock was prepared in 100% DMSO. The enzyme–inhibitor (1:5 molar concentration) complex was prepared on ice and kept for a minimum of 30 min before vitrification as described below. The final sample concentrations used for the preparation of grids were 0.043 and 0.025 mg ml^−1^ for the apo and atorvastatin-bound enzymes, respectively.

### 2.2. Cryo-EM grid preparation and data collection

Quantifoil Cu 300 mesh R1.2/1.3-type grids were covered with layers of graphene oxide (GO) as follows. Graphene oxide as a 2 mg ml^−1^ stock solution in water was purchased from Sigma (product No. 763705-25ML). 5 µl GO stock was diluted in 500 µl of a 5:1 methanol:water mixture. The methonal:water mixture has been shown to disperse the GO as monolayers better than using water alone as the solvent (Palovcak *et al.*, 2018 ▸; Kumar *et al.*, 2021 ▸). The methanol:water mixture-diluted GO solution was then centrifuged for 10 min and the supernatant was discarded. The remaining GO pellet was resuspended again in 500 µl methanol:water mixture and used to coat the grids. 5 µl of this solution was applied to the glow-discharged holey carbon support side of Quantifoil grids and left for 2 min for the GO flakes to be adsorbed. The excess solution was blotted away using a piece of filter paper through the bottom side and the grids were then air-dried. These grids were directly used for the vitrification of samples. Briefly, 5 µl sample was applied to the GO side and incubated for a minimum of 30 s for the sample to adsorb onto the GO. The grid was then manually blotted on the bench and immediately washed with 5 µl glycerol-free buffer. Subsequently, 3 µl glycerol-free buffer was applied onto the grid before the tweezers were latched onto a Thermo Fisher Scientific Vitrobot Mark IV. The grid was blotted using a blotting time of 8–9 s at a blot force of 10. The blotted grid was immediately plunged into liquid ethane for rapid vitrification and was stored in liquid nitrogen until screening and data collection on a microscope.

Single-particle cryo-EM data for the atorvastatin-bound sample were first collected on a Titan Krios G3i microscope (Thermo Fisher Scientific) equipped with a BioQuantum energy filter and a K3 direct electron detector (Gatan) at the electron Biological Imaging Center (eBIC) at Diamond Light Source (Table 1 ▸). Data acquisition was performed with the Thermo Scientific *EPU* software using a defocus range between −2.0 and −0.8 µm in steps of 0.2 µm with three exposures per selected hole at a magnification of 105 000×, corresponding to a pixel size of 0.83 Å. A total dose of 43 e^−^ Å^−2^ over 5 s exposure was fractionated into movies of 40 frames with a dose rate of 5.92 e^−^ pixel^−1^ s^−1^ in CDS bin2 mode (Table 1 ▸). A C2 aperture of 50 µm and an objective aperture of 100 µm were used.

The apoenzyme data were collected a few months later using the same microscope, now equipped with an F4i detector and a SelectrisX energy filter (Table 1 ▸). The magnification used was 130 000×. corresponding to a pixel size of 0.925 Å. Electron-event representation (EER) movies, each with 2151 internal frames, were recorded at a dose rate of 6.7 e^−^ pixel^−1^ s^−1^ over a 7 s exposure. The defocus range and aperture settings were the same.

### 2.3. Data processing, model building and validation

All data processing was carried out in *RELION* (Scheres, 2012 ▸; Kimanius *et al.*, 2021 ▸, 2024 ▸). Movies were imported and motion-corrected with *RELION* followed by *CTFFind* (Rohou & Grigorieff, 2015 ▸) to calculate the CTF parameters. *Topaz* (Bepler *et al.*, 2019 ▸) was first trained on a set of manually picked particles to make an optimized model and the model was then applied to the entire data set to pick particles. Initially, picked particles were extracted with a box size of 120 pixels with pixel size binned by 2. The particles set was split into six and four subsets for the apo and inhibitor-bound data, respectively, and two rounds of reference-free 2D classification were performed for each set. Particles belonging to class averages with well defined structural features were pooled together and an initial model was generated for use as a reference volume. The pool was then classified in 3D into three different class volumes and the class volume with well defined secondary-structural as well as tertiary-structural features was selected for further processing with unbinned data. For the final set of particles, one round of CTF refinement and Bayesian particle polishing was performed before the last step of refinement (Supplementary Figs. S1 and S2).

The final map was post-processed in *locspiral* (Kaur *et al.*, 2021 ▸) to increase interpretability and the resultant map was used for model building in *Coot* (Emsley *et al.*, 2010 ▸). The crystal structure model of hHMGR (PDB entry 1hwk; Istvan & Deisenhofer, 2001 ▸) was used as a starting model and was first rigidly fitted into the final map. A few rounds of manual model building in *Coot* and model refinement against two half-maps in *Refmac Servalcat* (Yamashita *et al.*, 2021 ▸) from *Doppio* (Burnley *et al.*, 2017 ▸) were carried out. At these resolutions of the cryo-EM map density for potential water molecules can be seen (Supplementary Fig. S3); however, they have not been modelled. The final built model was validated against the half-maps using the available *CCP-EM* pipeline tools in *Doppio* (Burnley *et al.*, 2017 ▸). The figures were prepared in *ChimeraX* (Meng *et al.*, 2023 ▸) using *locspiral*-sharpened maps.

## 3. Results and discussion

The purchased enzyme was at a low concentration of 0.214 mg ml^−1^ and this precluded the preparation of holey carbon EM grids. Therefore, we adopted the following procedure to prepare suitable grids for SPA cryo-EM. Firstly, the holey carbon layer of Quantifoil (Cu 300 mesh R1.2/1.3) grids was coated with graphene oxide (GO) flakes (see Section 2.2) to act as a support over the hole to increase the particle density. Secondly, to reduce the high background contrast from the glycerol in the buffer component and additionally from DMSO for the atorvastatin-bound sample, the grid was washed with buffer lacking glycerol prior to vitrification. Overall, our approach increased the contrast in the images sufficiently to obtain near-atomic resolution reconstructions of apo hHMGR and the atorvastatin-bound complex at 2.1 and 2.3 Å resolution, respectively, from particles adopting a slightly preferred orientation (Table 1 ▸; Supplementary Figs. S1 and S2).

**Figure 1:**
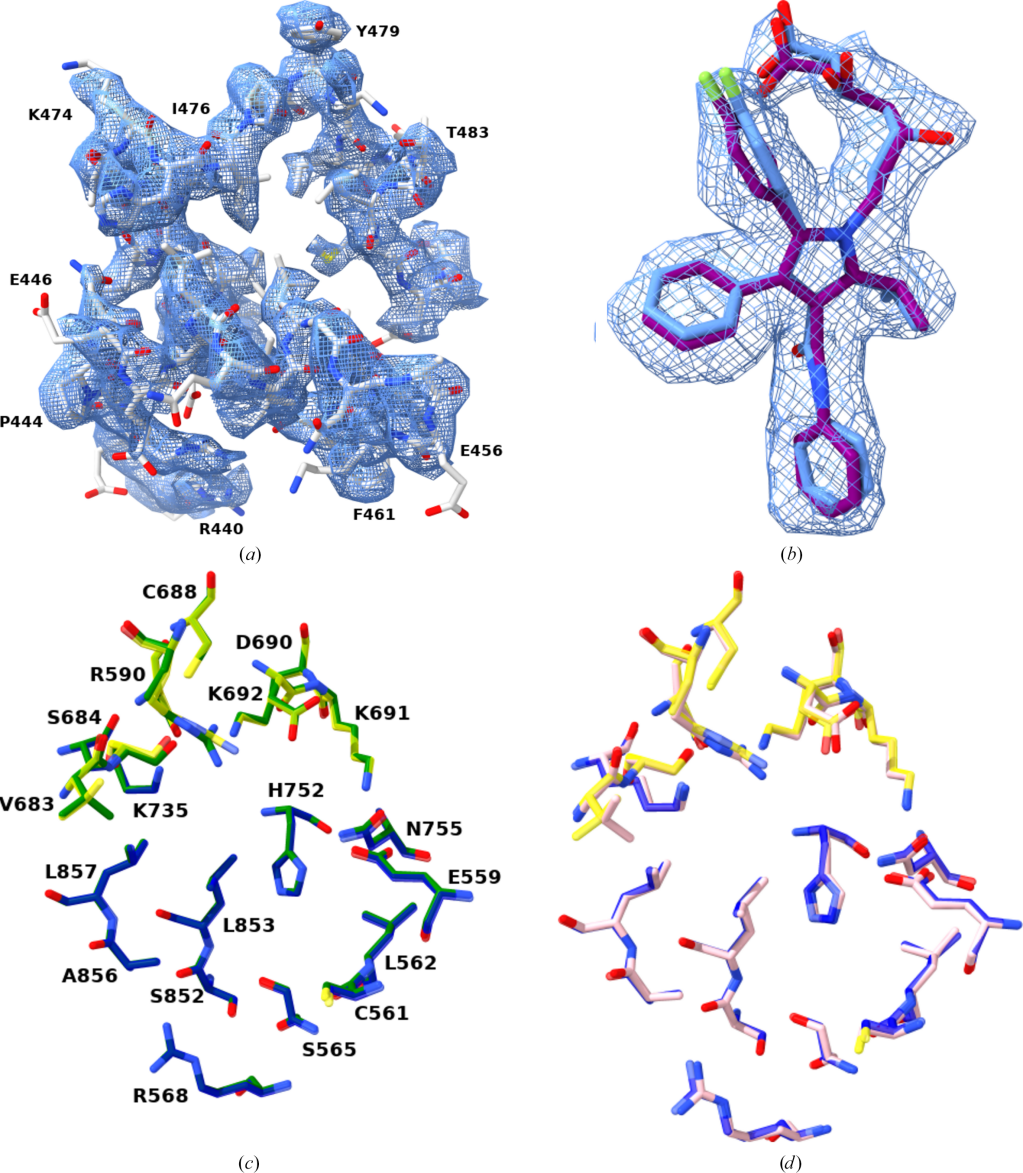
(*a*) The flexible N-domain. The commonly disordered region, which is part of the N-domain (residues 439–487) of the enzyme, is ordered in the cryo-EM structures and is modelled completely. The built model of the region is shown with side chains in ball-and-stick representation and coloured white. The *locspiral*-sharpened cryo-EM map is shown at a threshold level of 6 (for *ChimeraX*; volume zone nearAtoms range of 4.5 Å) in cornflower blue in mesh representation and several residues along the chain are labelled. (*b*) Atorvastatin density. The cryo-EM map at a threshold level of 3.5 (for *ChimeraX*; volume zone nearAtoms range of 3 Å) of atorvastatin is coloured in cornflower blue in mesh representation. The atorvastatin model is rendered in ball-and-stick representation (in cornflower blue for cryo-EM and in purple for the crystal structure PDB entry 1hwk). The atorvastatin binding seen in the cryo-EM sample is almost identical to the crystal structure conformation. (*c*, *d*) Active-site residues around atorvastatin. All residues in close contact with the inhibitor are shown in ball-and-stick representation. The corresponding residues in the apo enzyme are shown in green (*c*) and are partially transparent in the atorvastatin-bound structure (the residues belonging to two different monomers are shown in yellow and blue). The residues from the crystal structure (PDB entry 1hwk) are depicted in pink (*d*). For clarity, the residues are only labelled in (*c*) and the labelling holds true for (*d*). No noticeable change can be seen in the conformation of residues due to binding of the inhibitor to the active site.

As shown previously for the atorvastatin-bound form (Istvan *et al.*, 2000 ▸; Istvan & Deisenhofer, 2001 ▸) and from the apo structure in this report, the catalytic part of hHMGR forms a homotetramer comprising two homodimers arranged in *D*_2_ symmetry. The two neighbouring monomers of the dimer contribute residues to form an active site and hence there are four active sites per tetrameric complex. In all crystal structures reported to date, a region of N-terminal N-domain residues 439–487 was found to be highly flexible and therefore was only partially modelled. Notably, in both the apo and atorvastatin-bound cryo-EM structures the entire region can be modelled as the density is clearer (Fig. 1 ▸*a*). This may be partly attributed to the fact that the binding of particles to the GO surface resulted in stabilization as well as in a reduction of the flexibility of the N-domain of the enzyme.

Atorvastatin occupied all four active sites of the tetramer in the expected locations (Figs. 1 ▸*b* and 2 ▸*a*). When the apo and inhibitor-bound structures are compared as a tetramer, the overall r.m.s.d. of C^α^ atoms is found to be a maximum of 0.4 Å, indicating that the binding of atorvastatin does not alter the conformation of the complex (Figs. 1 ▸*c* and 2 ▸*a*). In particular, no major differences are seen in the active-site residues between the atorvastatin-bound and apo structures (Fig. 1 ▸*c*). In addition, the atorvastatin binding seen in the cryo-EM structure is identical to the conformation found in the crystal structure (PDB entry 1hwk; Istvan & Deisenhofer, 2001 ▸; Figs. 1 ▸*b* and 1 ▸*d*).

**Figure 2:**
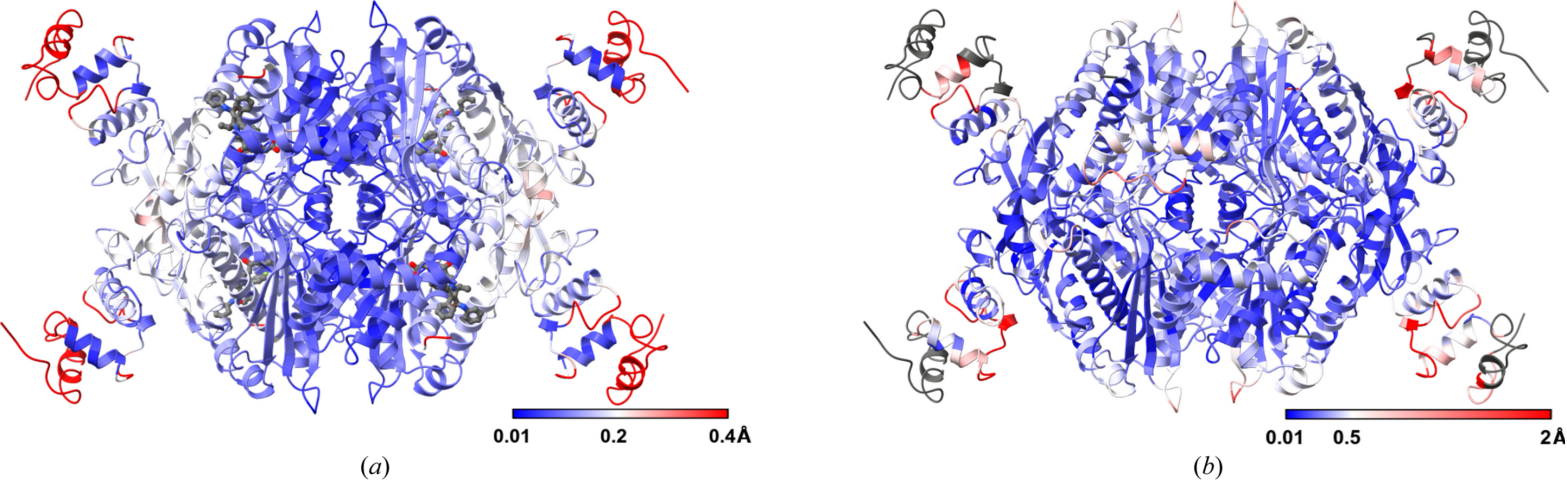
(*a*) Superposition of apo and atorvastatin-bound hHMGR cryo-EM structures. The atorvastatin-bound structure is shown in cartoon representation, coloured based on residue C^α^ deviations calculated against the apo structure on a linear blue–white–red scale with increasing magnitude. The maximum deviation is observed for the N-terminus of the N-domain. Atorvastatin bound in the four different active sites is also shown in ball-and-stick representation in grey. (*b*) Comparison of the crystal and cryo-EM structures of atorvastatin-bound hHMGR. The atorvastatin-bound cryo-EM structure (PDB entry 8pkn) is coloured based on residue C^α^ deviations calculated against the atorvastatin-bound crystal structure (PDB entry 1hwk) in blue–white–red with increasing magnitude. Apart from the loop regions being flexible, the N-domain, which functionally connects the catalytic portion of the enzyme to the membrane domain, is found to have relatively high deviations. The regions coloured in grey are those without equivalent residues in the crystal structure for comparison.

The comparison of crystal (PDB entry 1hwk) and cryo-EM (PDB entry 8pkn) structures of hHMGR confirms the inherent flexibility of the N-terminal N-domain as it has relatively higher C^α^ deviations (Fig. 2 ▸*b*). Further, the region has a lower local resolution compared with other parts of the protein (Supplementary Figs. S1 and S2). The described procedure of preparing cryo-EM grids using graphene oxide as an additional support for a sparse sample with high-contrast components in the buffer is expected to help to solve the structures of pharmaceutically important complexes by SPA cryo-EM.

## Supplementary Material

PDB reference: hHMGR, atorvastatin-bound, 8pkn

PDB reference: apo, 8s6b

EMDB reference: hHMGR, atorvastatin-bound, EMD-17748

EMDB reference: apo, EMD-19757

Supplementary Figures. DOI: 10.1107/S2053230X25001098/ih5007sup1.pdf

## Figures and Tables

**Table 1:** Cryo-EM data-collection, refinement and validation statistics

	hHMGR (apo)	hHMGR + atorvastatin
Data collection		
Microscope	Titan Krios G3i	Titan Krios G3i
Voltage (kV)	300	300
Detector	F4i	K3
Energy filter, slit width (eV)	SelectrisX, 10	BioQuantum, 20
Imaging mode	Counted	Counted CDS bin2
Magnification (×)	130000	105000
Dose rate (e^−^ pixel^−1^ s^−1^)	6.7	5.92
Exposure time (s)	7	5
Electron dose (e^−^ Å^−2^)	54.8	43.0
Pixel size (Å) at the detector	0.925	0.83
Defocus range set (µm)	−2.0 to −0.8 in 0.2 steps	−2.0 to −0.8 in 0.2 steps
No. of movies	13644	18337
Processing and reconstruction		
Program	*RELION* v5	*RELION* v4
Particles	1066707	404712
Box size (pixels)	240 × 240	240 × 240
Symmetry imposed	*D*_2_	*D*_2_
Resolution (Å)	2.06	2.26
FSC threshold	0.143	0.143
Map-sharpening *B* factor (Å^2^)	−76	−74
Efficiency of orientation distribution (*cryoEF*): *E*_od_ (Naydenova & Russo, 2017 ▸)	0.75	0.77
Model composition		
Protein residues	1692	1692
Atorvastatin ligand molecules	—	4
Accession codes		
Map	EMD-19757	EMD-17748
Model	8s6b	8pkn
Refinement		
Program	*Refmac Servalcat*	*Refmac Servalcat*
Resolution (Å)	2.06	2.26
FSC average	0.83	0.86
R.m.s. deviations		
Bond lengths (Å)	0.007	0.009
Bond angles (°)	1.19	1.26
Validation		
*MolProbity* score	1.94	1.70
Clashscore, all atoms	4.89	5.11
Rotamer outliers (%)	3.86	1.48
Ramachandran plot		
Favoured (%)	96.44	95.72
Allowed (%)	3.32	4.28
Outliers (%)	0.24	0.0
