# Supplementary material for: Cryo-EM structures of apo and atorvastatin-bound human 3-hydroxy-3-methylglutaryl-coenzyme A reductase
Source: Acta Crystallogr F Struct Biol Commun. 2025 Feb 20;81(Pt 3):118–22. doi: 10.1107/S2053230X25001098 (PMC11866412; doi:10.1107/S2053230X25001098)
Supplement: Supplementary file 1 [file f-81-00118-sup1.pdf]

Acta Cryst F

STRUCTURAL BIOLOGY
COMMUNICATIONS

Volume 81 (2025)

Supporting information for article:

Cryo-EM structures of apo and atorvastatin-bound human HMG-CoA reductase

Manikandan Karuppasamy and Jason van Rooyen

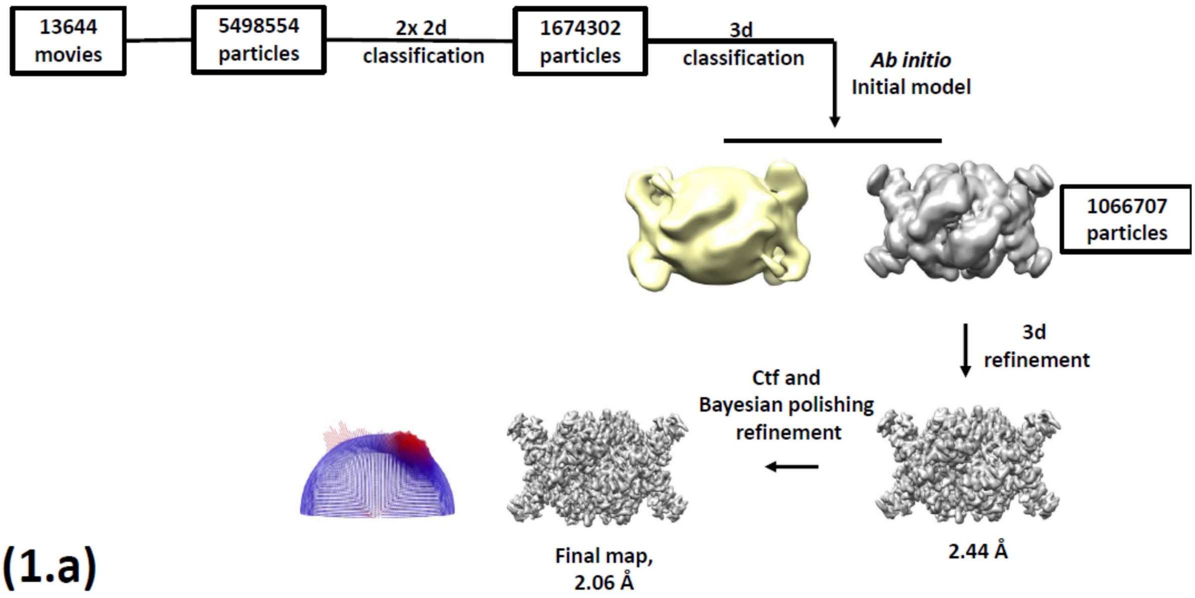
13644
movies
5498554
particles
2x 2d
classification
1674302
particles
3d
classification
Ab initio
Initial model
1066707
particles
3d
refinement
Ctf and
Bayesian polishing
refinement
2.44 Å
Final map,
2.06 Å

(1.a)

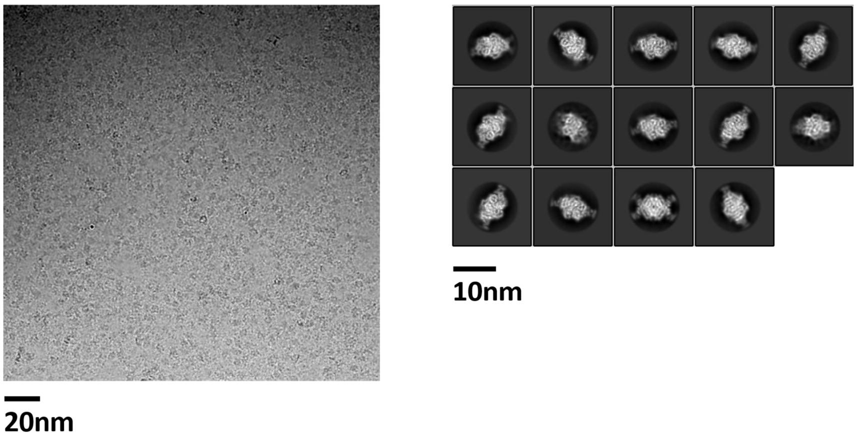
20nm
10nm

(1.b)

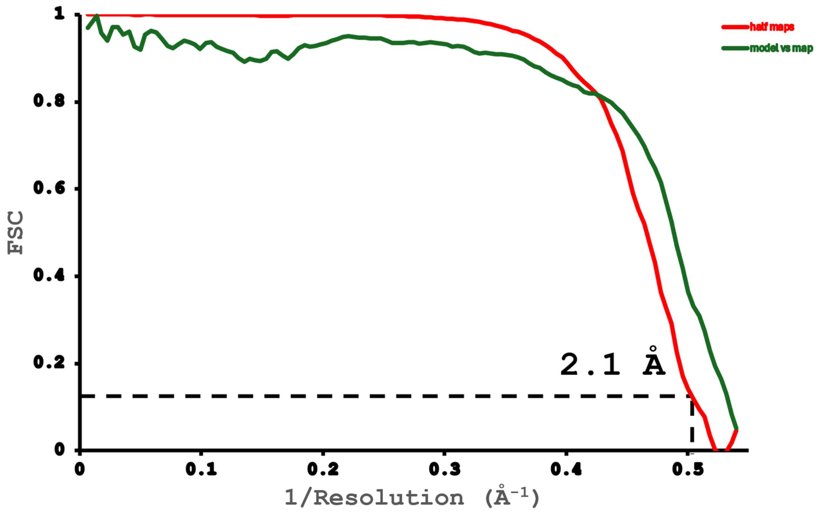

(1.c)

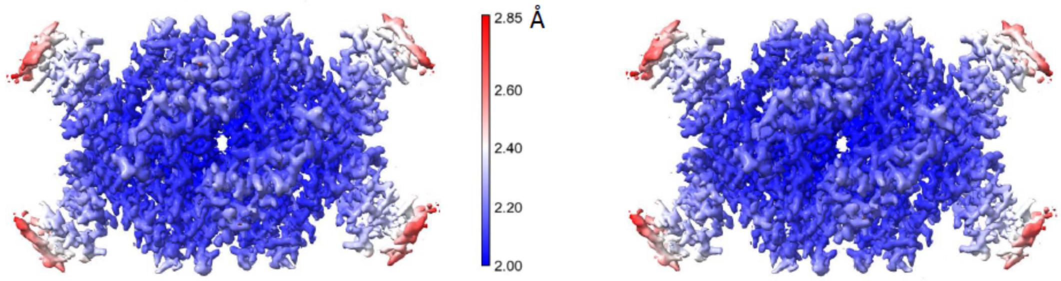

(1.d)

Figure S1 A schematic workflow and results of SPA cryo-EM data processing performed in *RELION* for the apo enzyme data. (a) Data processing steps carried out to arrive at final reconstruction. (b) A representative motion-corrected micrograph and selected reference-free 2D class averages. (c) A plot of half maps and model-to-map FSC calculated by *RELION* and *RefmacServalcat*, respectively. The model-to-map FSC values at 0.5 cut-off were 2.05 Å and 2.28 Å, respectively for apo and atorvastatin-bound data. (d) The final map coloured based on local resolution values computed in *RELION*.

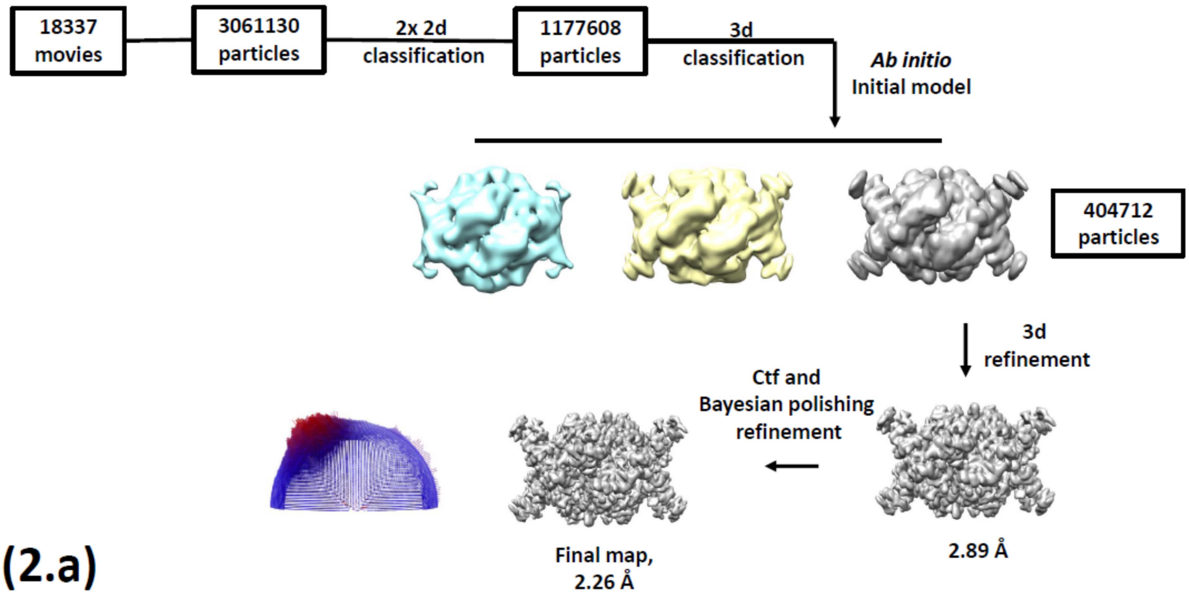
18337
movies
3061130
particles
2x 2d
classification
1177608
particles
3d
classification
Ab initio
Initial model
404712
particles
3d
refinement
Ctf and
Bayesian polishing
refinement
Final map,
2.26 Å
2.89 Å

(2.a)

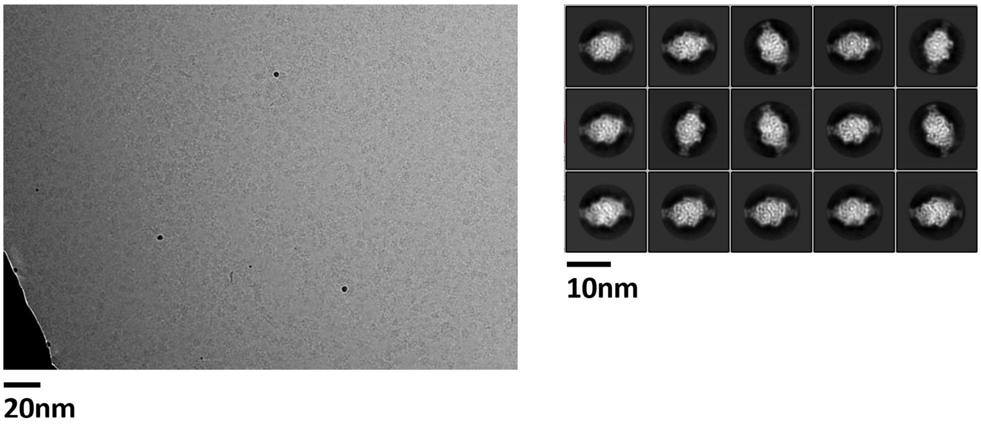
20nm
10nm

(2.b)

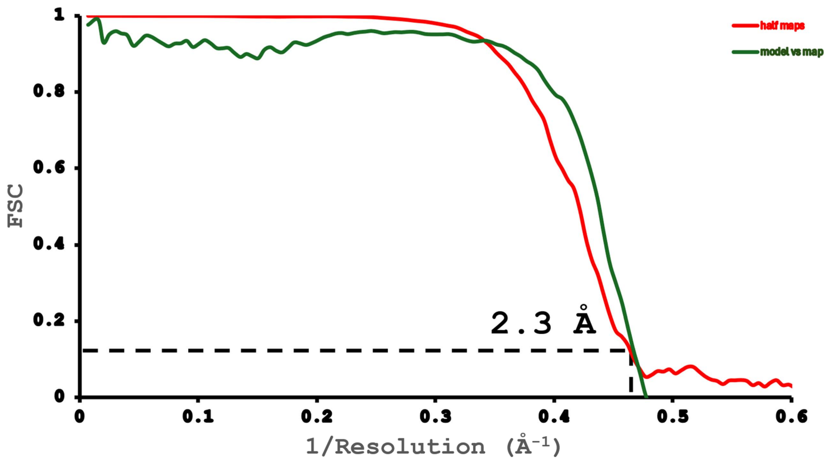

(2.c)

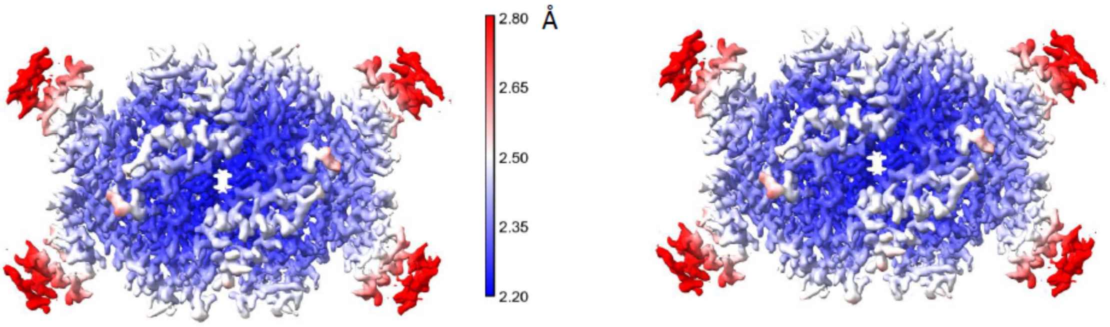

(2.d)

Figure S2 A schematic workflow and results of SPA cryo-EM data processing performed in *RELION* for the atorvastatin-bound data. (a) Data processing steps carried out to arrive at final reconstruction. (b) A representative motion-corrected micrograph and selected reference-free 2D class averages. (c) A plot of half maps and model-to-map FSC calculated by *RELION* and *RefmacServalcat*, respectively. The model-to-map FSC values at 0.5 cut-off were 2.05 Å and 2.28 Å, respectively for apo and atorvastatin-bound data. (d) The final map coloured based on local resolution values computed in *RELION*.

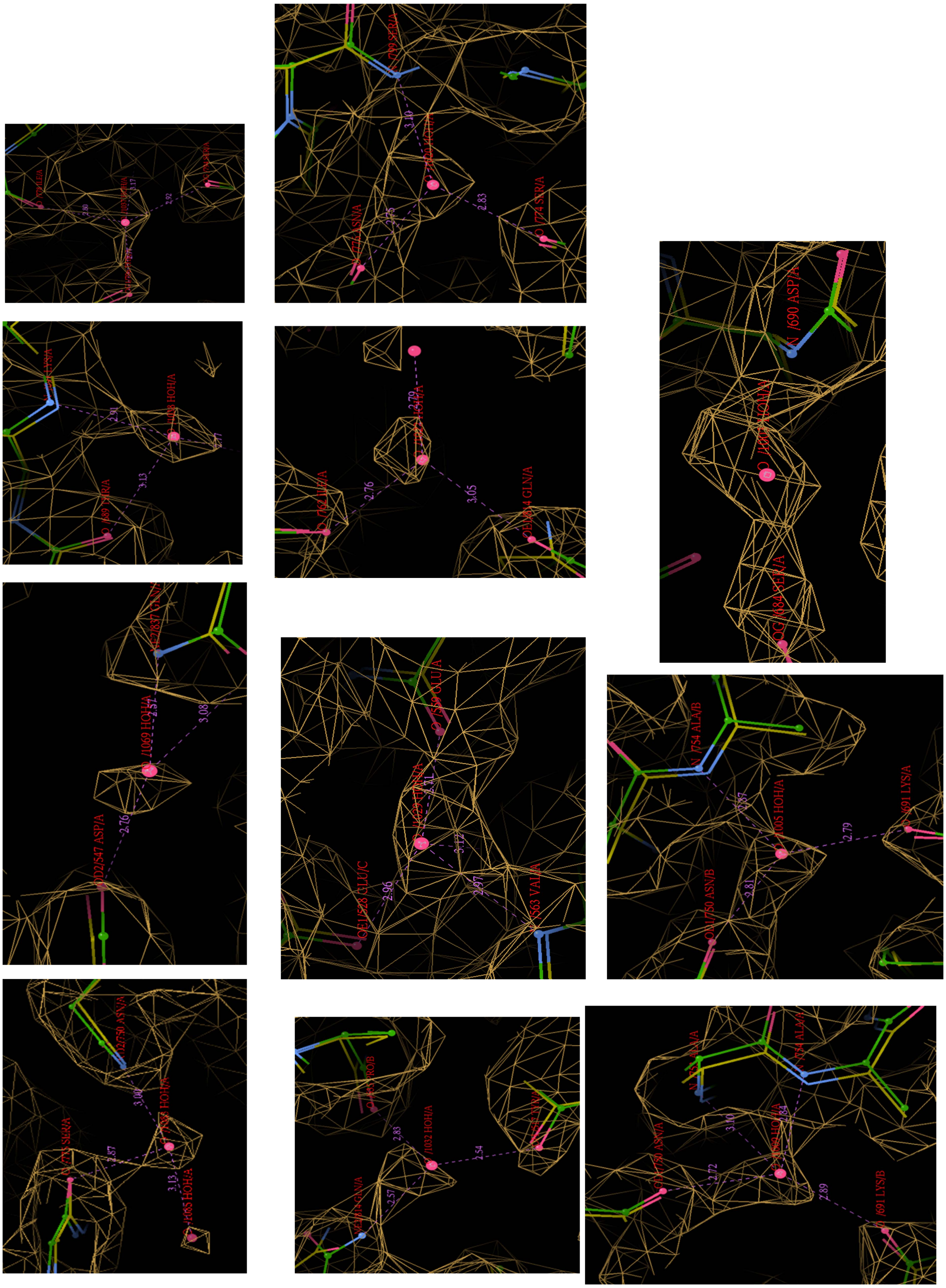

Figure S3 Water molecules can be located at these resolutions of the cryo-EM map. The crystal structure model, PDB-1hwk is superposed onto the cryo-EM map of atorvastatin-bound data along with the derived model, PDB-8pkn and both models are shown in stick representation. The cryo-EM

map of atorvastatin-bound data shown at 2σ threshold in *Coot* (in mesh representation in orange). The cryo-EM map shows clear density for water molecules at representative water molecule positions (as red spheres) that were found in the crystal structure.
